# Clinical effects and safety of electroacupuncture for the treatment of allergic rhinitis: A protocol for systematic review

**DOI:** 10.1097/MD.0000000000018931

**Published:** 2020-02-07

**Authors:** Li Fu, Juan Zhong, Qinwei Fu, Yepeng Yang, Mengni Zhang, Qinxiu Zhang

**Affiliations:** a Hospital of Chengdu University of Traditional Chinese Medicine; b School of Medical and Life Sciences/Reproductive & Women-Children Hospital, Chengdu University of Traditional Chinese Medicine, PR China.

**Keywords:** allergic rhinitis, clinical trial, electroacupuncture, protocol, systematic review

## Abstract

**Background:**

As the therapy of acupuncture continues to expand, electroacupuncture is increasing used in allergic rhinitis prevention and therapy, and many studies have published that it is effective in the treatment of allergic rhinitis. However, it is unclear if electroacupuncture provides an evidence-based clinical benefit in the allergic rhinitis population.

**Methods/design:**

We will conduct a systematic review of electroacupuncture and health-related outcomes in allergic rhinitis patients according to the Preferred Reporting Items for Systematic Reviews. The primary objective is to assess the impact of Preferred Reporting Items for Systematic Reviews and Meta-Analyses (PRISMA) guidelines on clinical outcomes relevant to allergic rhinitis patients, such as effective rate, life-quality evaluation, and adverse events. The Cochrane Risk Assessment Tool will be used to assess the quality of eligible studies according to the Cochrane handbook.

**Results:**

We will perform a meta-analysis using standard techniques for the outcomes.

**Conclusion:**

The results of this systematic review may highlight the gaps in our knowledge of the relevance of electroacupuncture after treatment or after follow-ups.

PROSPERO registration number: PROSPERO CRD42018104080. (https://www.crd.york.ac.uk/PROSPERO/#recordDetails)

## Abstract 2

Supplemental Digital Content is available in the text

## 1 Introduction

Allergic rhinitis (AR) poses a global health problem through the inducing of immunoglobulin E- (IgE)-mediated sensitization to environmental allergens. Over 25% of Swedish citizens had been affected today,^[1]^ with evidence suggesting that the prevalence of the disorder is increasing.^[2]^ Because of considerable symptomatic (nasal discharge, sneezing, nasal itching, and congestion) burdens, AR claims a high toll on patients’ lives, and negatively impacts patients’ quality of life (QoL),^[3–8]^ this may include performance at work and school,^[9,10]^ and can be associated with poor sleep quality,^[11]^ mood impairment,^[12]^ and even the ability to drive.^[13]^

Subcutaneous injection immunotherapy or sublingual immunotherapy are common treatment modalities for this disease, but the effectiveness of the therapies are in doubt and remain to be demonstrated conclusively.^[14]^ Other Conventional medical treatments are only moderately efficacious, and they often produce problematic side effects.

Acupuncture/electroacupuncture had been used in China and other Asian countries for the past 3000 years, which represents a potentially valuable adjunct to existing allergic rhinitis symptom relief strategies.^[15]^ Approximately two million American adults used acupuncture in 2002,^[16]^ this increased to three million in 2007. Electroacupuncture, a modification of conventional acupuncture therapy, is an electrical stimulation administered through acupuncture needles. It is believed that electrical impulses could strengthen the stimulation from the needles at the acupoints. Human body tissue used as an electrical conductor, hence, electrical stimulation can generate directional movement of ions and eliminate polarization of the cell membrane.

A number of studies provided evidence as a beneficial effect of acupuncture on AR.^[17–20]^ Although it seems that it is rational to use electroacupuncture for AR, further investigation in a series of clinical trials is required to draw a reliable conclusions about the effectiveness and safety of this treatment.

## 2 Methods/design

### 2.1 Research objectives

We will conduct a systematic review to determine the clinical efficacy and safety of electroacupuncture on the outcomes of allergic rhinitis patients (presenting with seasonal allergic rhinitis or perennial allergic rhinitis).

### 2.2 Inclusion criteria for study selection

#### 2.2.1 Types of studies

Only randomized, controlled trials (RCTs) of electroacupuncture for any age of AR patients will be included in this systematic review. It means that prospective randomized controlled trials (RCTs) of electroacupuncture versus a placebo regimen or conventional medicine regimen will be included. If we are unable to find at least five eligible RCTs for the systematic review, we will broaden our inclusion criteria to include semi-randomized control studies, non-randomized studies of electroacupuncture in AR patients using the Cochrane Effective Practice and Organization of Care (EPOC) approach to categorize the types of studies.^[21]^ We acknowledge that the brand of electroacupuncture and the quantity of stimulus will vary among studies, but in order to maximize the total study population, we will not require one particular type or stimulus schedule.

#### 2.2.2 Types of patients

Patients who meet the diagnostic criteria of AR, either presenting with seasonal AR or perennial AR were all included. However, AR merged with allergic asthma or allergic conjunctivitis and other allergic diseases were excluded. This was done because targeted drug combination methods in these studies could not be used to compare the effects. All included participants in this review regardless of their age, race, and gender.

#### 2.2.3 Study of exclusion criteria

All observational studies, case reports, case series, qualitative studies, uncontrolled studies, and other study designs for which there are no randomization control methods will be excluded. All animal studies will be excluded. We will also exclude studies involving patients with allergic rhinitis merging with allergic asthma or allergic conjunctivitis and other allergic diseases, because the targeted drug combination methods applied could result in researchers not being able to determine effects.

#### 2.2.4 Types of interventions and controls

We will only include studies which interventions involved electroacupuncture with conventional medicine or placebo regimens. However, studies that compare the efficacy of different forms of electroacupuncture will be excluded. Interventions considered for experimental groups vs control groups were as follows:

1. Electroacupuncture vs conventional medicine,
2. Electroacupuncture combined with conventional medicine vs conventional medicine.
3. Electroacupuncture combined with other complementary therapies vs other complementary therapies.
4. Electroacupuncture vs placebo or no therapy
5. Electroacupuncture vs pseudo-electroacupuncture therapy or no therapy

We excluded studies or trials with electroacupuncture performed as a part of complex interventions versus other types of regimens, for example, electroacupuncture plus another herbal medicine formula vs acupuncture therapies.

#### 2.2.5 Types of outcomes

The pre-specified primary outcomes will be the improvement of AR symptoms, which will include an effective rate result, or any related scale or score, such as the Nasal symptom score, or Improvement in quality of life. The secondary outcomes will include results, such as adverse events, serum level change, or recurrence rate. These pre-defined outcomes may be adjusted during the review, depending on the outcomes identified in eligible studies.

### 2.3 Search methods for the identification of studies

#### 2.3.1 Data sources

We will employ a broad electronic search strategy. PubMed, EMBASE (Excerpta Medical Database), The Cochrane Library, the Chinese Cochrane Centre's Controlled Trials Register platform, the Wanfang Chinese digital periodical and conference database, the China National Knowledge Infrastructure (CNKI) database, and the VIP Chinese Science and Technique Journals Database will be researched by our author for relevant literature. In addition, the Chinese Clinical Trial Registry Center will also be screened for ongoing trials. We will also review the references of included manuscripts to identify any information about missed trials. We will contact the author if we cannot clearly identify information from the data.

#### 2.3.2 Search strategy

We will employ a broad electronic search strategy in Supplemental Digital Content (Appendix A).

### 2.4 Data extraction, quality and validation

#### 2.4.1 Study screening and inclusion

All abstracts returned using the search strategy above will be screened by 2 independent investigators (LF, YPY) in line with our advanced inclusion criteria. And then, the full text of the entire study will be reviewed by three authors for analysis. Researchers will import the literature retrieved to the Endnote X7 and eliminate the duplicate data. The final list of articles will be converted into Microsoft Excel format.

#### 2.4.2 Data extraction

The raw data from the papers will be extracted separately by 3 authors and will include: author details, publication information, sample size, and original study design information. Electroacupuncture brand and the quantity of stimulus information will be also extracted by us. All extracted data will be verified by a second investigator to ensure accuracy and completeness. All outcome variables will be collected, regardless of the number of studies that the outcome assessed.

### 2.5 Quality assessment/Risk of bias

The methodological quality for the included RCTs will be assessed based on the instrument developed in the Cochrane Handbook for Systematic of Interventions. The tool evaluates studies based on seven criteria:

1) randomization generation,
2) allocation concealment,
3) blinding of outcome assessors,
4) blinding patients/study personnel,
5) incomplete outcome data (that is, lost to follow-up),
6) selective outcome reporting, and
7) other risks of bias.

We will define other bias as trials which may be sponsored by electroacupuncture experts, and in which baseline characteristics are not similar between the different intervention groups. We will also assess publication bias by examining funnel plots if there are 10 or more trials reporting the primary outcomes.

### 2.6 Quantitative data synthesis and statistical methods

#### 2.6.1 Quantitative data synthesis

**Figure 1:**
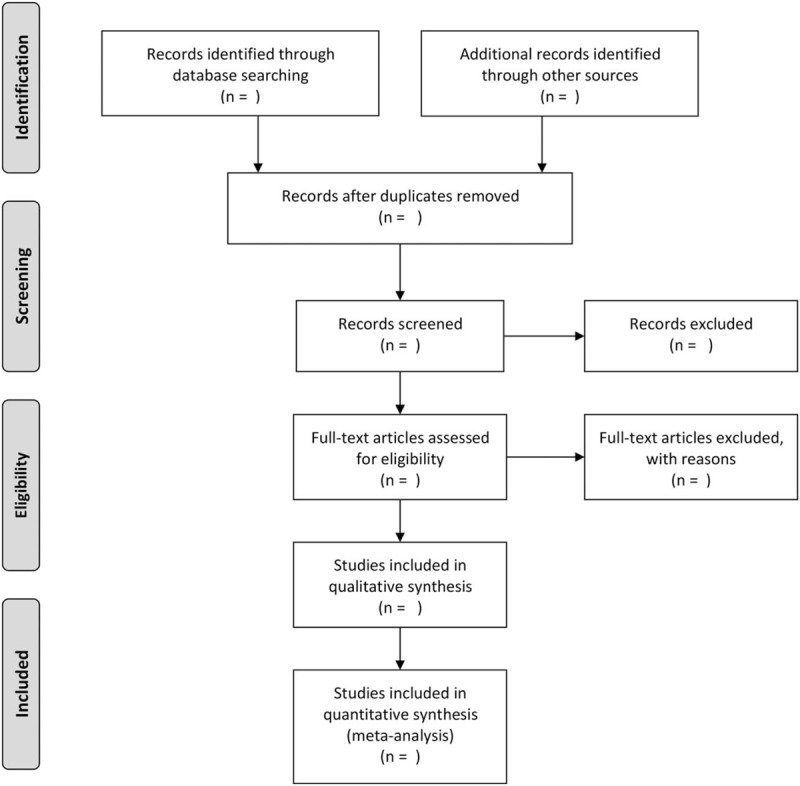
Flow diagram of study selection process.

A Preferred Reporting Items for Systematic Reviews and Meta-Analyses diagram (Fig. 1) based on the search strategy and eligibility assessment to show the flow of included and excluded studies will be developed by us.^[22]^ Review Manager (RevMan) software version 5.3 will be applied to pool our data to perform the meta-analysis. The measurements of dichotomous data will to be expressed as relative risks along with 95% confidence intervals (CIs); for continuous data, mean difference, 95% CIs will be adopted, and *P* < .05 will be defined as statistically significant. Figure 1 PRISMA diagram

#### 2.6.2 Assessment of heterogeneity

In this review, I^2^ values will be used to assess inter-study heterogeneity. When I^2^ > 75%, considerable heterogeneity will be conformed, whereupon a random effects model will be applied. We will pool trials when the intervention form of those studies is adequately similar. We will pool treatment efficacy/effective rate estimates when using standard statistical techniques and RevMan software. Adverse events will be evaluated as well as. Additional outcomes such as Visual analogue score (VAS), Quality of life inventory for nasal conjunctivitis (RQLQ), and other symptom scoring system results as recommended by guidelines,^[23]^ will be also analyzed. Depending on the heterogeneity of quantity of stimulus schedules, we may also perform separate analyses for differing quantity of stimulus regimens. Finally, as follow-up time may vary from trial to trial, we will include time as a covariate in our final data analysis.

#### 2.6.3 Assessment of reporting bias

If a sufficient number of studies are available (at least 10 studies), we will attempt to assess publication bias using a funnel plot.

#### 2.6.4 Subgroup analysis and investigation of heterogeneity

If there is a significant heterogeneity in the included trials, we will conduct subgroup analysis based on the sub-type of disease, differences in treatment frequencies and follow-up duration will also be included.

#### 2.6.5 Sensitivity analysis

If the test for heterogeneity *P* value is less than .1 after performing the subgroup analysis, the sensitivity analysis will be conducted to evaluate the robustness of our results. The meta-analysis will repeated after omitting the low-quality studies. Moreover, we will also assess whether the statistical model (random-effects vs. Fixed-effects model) will affect the current results.

#### 2.6.6 Grading the quality of evidence

We will apply the Grading of Recommendation Assessment, Development and Evaluation (GRADE) method to evaluate the level of confidence in regards to outcomes. Two independent reviewers will conduct the assessment. In most cases, disagreement was resolved by discussion. If disagreement remained after discussion, a third reviewer will be consulted before taking the final decision on the disagreement.

## 3 Discussion

Acupuncture therapy was recommended by clinical guidelines,^[24]^ while guidelines do not address electroacupuncture as a routine therapy in AR patients, even electroacupuncture with the similar function and effect mechanism compared to acupuncture therapy.^[20]^ Perhaps this is due to the lack of depth specific clinical effect or effect mechanism research. However, emerging evidence indicates that the role electroacupuncture may play in human health that can significantly ameliorat inflammatory and nociceptive mediators both peripherally and centrally in sickle mice correlative to the antinociceptive response.^[25]^ As such, determining the optional functional therapy of allergic rhinitis patients is of vital importance and is the purpose of the proposed systematic review.

We foresee several potential limitations with this systematic review: heterogeneity of clinical outcomes, substandard quality of existing studies, which are the focus of our project. Therefore, we will present our findings using descriptive methods, if necessary. This study protocol has been designed according to conventional acupuncture therapy for treatment of allergic rhinitis based on the study data or outcomes from existing published (and non-published) literature. Our hope is that the dissemination of this protocol will allow us to obtain feedback and constructive criticism of the methods before our study is conducted.

In conclusion, the proposed systematic review will provide insight into the clinical impact of electroacupuncture in treatment of allergic rhinitis patients. The results have the potential to inform national and international guidelines on the care and management of electroacupuncture in the allergic rhinitis population. The review will also help to highlight areas requiring further research on this topic.

## Author contributions

LF, JZ and QXZ conceived the study, designed the review, and wrote the initial manuscript. QWF, YPY and MNZ performed the initial searches to determine the feasibility, provided input into the study design, and reviewed the manuscript. All authors read and approved the final manuscript.

## Supplementary Material

Supplemental Digital Content
