# Supplementary material for: Clinical effects and safety of electroacupuncture for the treatment of allergic rhinitis: A protocol for systematic review
Source: Medicine (Baltimore). 2020 Feb 7;99(6):e18931. doi: 10.1097/MD.0000000000018931 (PMC7015649; doi:10.1097/MD.0000000000018931)
Supplement: Supplemental Digital Content [file medi-99-e18931-s001.doc]

The search strategy will be:

#1 electrical acupuncture

#2 electroacupuncture

#3 (electric AND (acupuncture OR needle OR acupoint OR point OR stimulat))

#4 #1~#3/OR

#5 allergic rhinitis

#6 rhinallergosis

#7 AR

#8 #5~#7/OR

#9 #4 AND #8
